# Interprofessional education in palliative care: a theory-informed evaluation of a storyline-based curriculum for health and social care students

**DOI:** 10.1186/s12909-026-10663-3

**Published:** 2026-10-22

**Authors:** Kambiz Afshar, Franziska Grimm, Kirsten Kopke, Anja Goral, Sven Schwabe

**Affiliations:** 1 Institute for General Practice and Palliative Care, Hannover Medical School, Carl-Neuberg-Straße 1, Hannover, 30625 Germany; 2 Department of Nursing and Health Care, Faculty V, Diaconic Studies, Health Care and Social Work, Hochschule Hannover - University of Applied Sciences and Arts, Hannover, Germany; 3 Department of Religious Education and Protestant Church’s Welfare Services, Faculty V - Diaconic Studies, Health Care and Social Work, Hochschule Hannover - University of Applied Sciences and Arts, Hannover, Germany

**Keywords:** Interprofessional education, Palliative care, Professional role, Attitude, Social identity, Students, Communication

## Abstract

**Background:**

Interprofessional education (IPE) in palliative care is essential for fostering collaborative, patient-centred practice. However, existing programs often underrepresent non-medical professions and insufficiently address spiritual care, ethical reflection, and experiential learning, despite the relevance of these aspects to comprehensive, holistic palliative care. This study evaluates an innovative interprofessional curriculum integrating the Canadian Interprofessional Health Collaborative (CIHC) framework, Social Identity Theory (SIT), and Transformative Learning Theory (TLT), designed to promote holistic, values-based learning through narrative-based, experiential methods.

**Methods:**

A mixed-methods design was employed in the second implementation cycle (summer term 2025) of the course ‘*Interprofessional Collaboration in Palliative Care*’ at Hochschule Hannover, involving 25 students from medicine, nursing, social work, and religious education. A quantitative survey assessed knowledge, attitudes, and skills, while two focus groups explored learning experiences and interprofessional collaboration within the course. Data were analysed using descriptive statistics and inductive-deductive qualitative content analysis (Kuckartz & Rädiker) with MAXQDA 2024.

**Results:**

A total of 19 students participated in the survey, and 10 students took part in the two focus groups. Students reported high satisfaction with the course structure, practical orientation, and the storyline method, which facilitated engagement and team-based learning. The survey showed particularly positive ratings for social and communication skills, knowledge of other professional groups, and team-oriented problem solving. Qualitative analysis revealed eleven sub-codes allocated to three deductive main codes: (1) the course’s narrative design enabled deep reflection on professional identity and ethical responsibilities; (2) underrepresentation of medical and nursing students in some groups risked professional hierarchies; (3) students expressed a strong desire for more balanced professional representation, discipline-specific content, and clinical experiences, such as visits to hospices or contact with patients.

**Conclusions:**

This study demonstrates that a theoretically grounded, narrative-based IPE curriculum can support interprofessional competence and reflection on professional roles in palliative care. However, challenges remain in ensuring equitable professional representation and integrating authentic clinical experiences. Future programs must reflect time limitations, balance narrative engagement with discipline-specific depth and integrate structured clinical placements or simulated patient encounters to fully prepare students for the complex, collaborative realities of palliative care practice.

**Supplementary Information:**

The online version contains supplementary material available at https://doi.org/10.1186/s12909-026-10663-3.

## Background

### Clinical and educational context

Delivering high-quality palliative care requires coordinated collaboration among multiple professional groups, as patients and families often face complex physical, psychosocial, and spiritual needs [1]. Palliative care aims to improve the quality of life of patients and their families facing life-threatening illness by addressing these multidimensional needs and preventing and alleviating suffering [1]. Palliative care is grounded in values of dignity, compassion, autonomy, and holistic support, emphasising not only symptom management but also communication, ethical reflection, and involvement of families and significant others [2]. No single profession can meet all needs of patients and relatives. Effective interprofessional collaboration is essential but remains challenging due to differing professional cultures, hierarchies, and communication barriers [3]. For students preparing to work in palliative care, developing interprofessional competencies alongside profession-specific knowledge and skills is therefore important. Interprofessional education (IPE) has gained increasing attention as a strategy to strengthen collaborative practice through shared learning experiences across professional boundaries.

### Existing research on IPE in palliative care

Numerous IPE initiatives in palliative care have been implemented across undergraduate and postgraduate levels, typically demonstrating improvements in students’ knowledge, communication skills, and attitudes toward teamwork [4–7]. However, the professional composition of these initiatives remain narrow: a recent systematic review of interprofessional palliative care education programs found that among 39 studies, learners from medicine and nursing were most frequently included, while disciplines such as social work, spiritual care, or psychosocial professions were markedly underrepresented [4]. This pattern is not specific to palliative care: a broader systematic review of IPE in health and social care education similarly identified underrepresentation of non-medical and non-nursing professions and called for more inclusive, multisectoral program designs [8]. Spiritual care in particular is often insufficiently addressed in interprofessional education programs, despite its recognised centrality to palliative care: the European Association for Palliative Care (EAPC) White Paper emphasises that spiritual care should be systematically incorporated into educational activities, training models, and competency frameworks to ensure holistic, patient-centred care across all professional groups [9]. Beyond disciplinary representation, few studies to date have examined how interprofessional learning in palliative care facilitates personal transformation, challenges professional stereotypes, or supports the development of shared professional identity. Taken together, these gaps point to the need for innovative, theoretically grounded educational models that capture the emotional, ethical, and relational complexity of palliative care practice and better prepare students for collaborative practice across professional boundaries.

### Theoretical framework

To provide a coherent conceptual basis for the design and evaluation of the interprofessional learning intervention, we integrated three complementary perspectives that address different but interconnected levels of interprofessional learning. The competency framework of the Canadian Interprofessional Health Collaborative (CIHC) [10–12] provides a macro-level perspective by defining the competencies that IPE aims to develop. Social Identity Theory (SIT) [13] offers a meso-level perspective by explaining how professional group identities and intergroup dynamics can shape interprofessional collaboration. Transformative Learning Theory (TLT) [14] provides a micro-level perspective by focusing on changes in learners’ individual assumptions, perspectives, and professional self-understanding. This three-level structure is used as an analytical framework for the present study, linking the competencies to be developed with the social processes and individual learning processes through which interprofessional learning may occur.

#### Macro level: competency frameworks (CIHC)

The development of interprofessional competencies has been guided by structured frameworks that inform curriculum design and evaluation. The CIHC framework [10, 11] delineates six essential domains that serve as pedagogical anchors for effective IPE: patient-centred care, role clarification, interprofessional communication, team functioning, collaborative leadership, and conflict resolution [12]. The framework has previously been applied to examine collaborative practice in healthcare settings, where it helped to identify both strengths and areas for improvement across these competency domains [12]. In the present study, the framework therefore provided a basis for structuring the learning objectives and evaluating students’ development of interprofessional competencies. These competencies are highly relevant for palliative care, where team-based decision-making and relational sensitivity are fundamental.

#### Meso level: professional identities and social dynamics (SIT)

Interprofessional learning must also address the social dynamics that shape intergroup relations. SIT [13] provides a lens to understand how professional identities can both enable and constrain teamwork. When healthcare students define themselves primarily by their discipline, in-group favouritism and out-group stereotypes may arise, impeding mutual respect and collaboration. IPE activities that foster positive intergroup contact and shared goals can counteract these effects and build a more inclusive professional culture. Khalili et al. [14] applied concepts from SIT and intergroup contact theory to an interprofessional socialisation framework, highlighting the development of a dual professional and interprofessional identity among health professions students. In the present study, this perspective informed the expectation that shared learning across professional groups would create opportunities to reflect on professional roles and develop interprofessional identity.

#### Micro level: personal transformation and reflection (TLT)

At a micro level, IPE can stimulate deep personal and professional reflection. TLT [15] emphasises that adult learning becomes transformative when learners critically examine and revise their underlying assumptions and beliefs. TLT has previously been applied to interprofessional learning simulation, where it provided a framework for exploring transformative learning processes and changes in participants’ perspectives and understanding of professional roles [16]. In the emotionally demanding field of palliative care, such reflection is particularly relevant: learners are invited to confront issues of suffering, death, and ethical decision-making, which may create opportunities for shifts in self-understanding and professional identity. In the present study, TLT therefore informed the expectation that the experiential and reflective elements of the course would provide opportunities for students to critically examine their assumptions and perspectives.

### Curriculum development and implementation

**Fig. 1:**
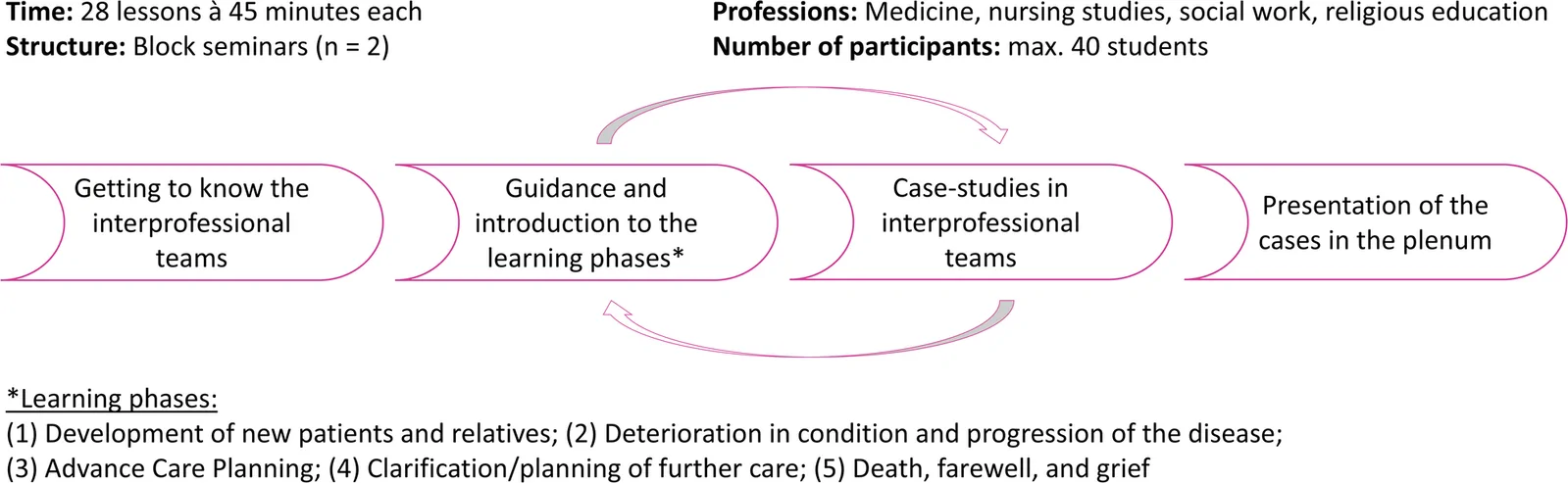
Structure and content of the course “Interprofessional Collaboration in Palliative Care”

Building on these conceptual foundations and addressing the identified research gaps, our interdisciplinary team – comprising experts in medicine, nursing, nursing sciences, social work, religious education, and social sciences from Hochschule Hannover and Hannover Medical School – developed an interprofessional curriculum entitled “*Interprofessional Collaboration in Palliative Care*” (see Fig. 1) [17]. The course brings together students from medicine, nursing, social work, and religious education to engage in scenario-based learning using the Storyline method [18–20], an approach that integrates creative narrative design with experiential, reflective, and collaborative learning. Within interprofessional groups, students co-create a fictional patient and accompany this person through key palliative care phases, including hospital admission, advance care planning, ethical decision-making, end-of-life care, and bereavement. The course programme included interactive lectures, exchanges with practitioners, small-group work, practical exercises (e.g., communication skills), and a final presentation, all structured around the overarching storyline method. Our curriculum operationalises these three levels: macro-level competencies guide team objectives, meso-level considerations address intergroup dynamics, and micro-level reflection fosters transformative learning. This structure aims to integrate cognitive, emotional, and social dimensions of interprofessional learning.

### Aim and objectives

The present study focuses on the second implementation cycle of the course (summer term 2025) and examines how participants’ knowledge, attitudes, and skills regarding palliative care and interprofessional collaboration developed through this learning experience. Furthermore, it explores how students’ reflections and group interactions illustrate the three theoretical perspectives introduced above (macro, meso, and micro levels). By linking empirical findings to established frameworks and addressing underexplored aspects of interprofessional learning in palliative care, this study seeks to contribute to the growing evidence base for theoretically grounded, value-oriented interprofessional education.

## Methods

### Study design

We adopted a mixed-methods design to explore participants’ perceptions of the course, focusing on the structure and design of the course, knowledge development, attitude formation, skill acquisition, and interprofessional collaboration. The mixed-methods design, including a survey and focus groups, was employed to provide a comprehensive evaluation of the course. It enables both a broad assessment of the course by a larger number of students and an in-depth exploration of the experiences underlying these assessments. The methodology and reporting followed the Consolidated Criteria for Reporting Qualitative Research (COREQ) [21].

### Setting

The course ‘*Interprofessional Collaboration in Palliative Care*’ was implemented in summer term 2025 for the second time with *n* = 25 students from medicine, nursing, social work and religious education [17]. The course was made available to students who were either at an advanced stage of their studies or who already had practical professional experience. The course was offered as an optional elective course in all degree programmes.

### Participants

At the end of the course, all 25 students were invited to participate in the quantitative online survey and in the focus groups. Participation in both components was voluntary. A schedule was subsequently arranged with all students who had consented to participate in focus groups. The research team then proceeded to establish the dates on which most of the participants were available and on which at least one person from each degree programme was represented. Furthermore, double entries were excluded. The research team consisted of KA (postdoctoral researcher, male, medicine and medical didactics), VB (professor, female, social work), AG (research associate, female, religious education), FG (research assistant, female, nursing sciences), KK (professor, female, nursing sciences), and SvS (postdoctoral researcher, male, social science).

### Data collection

The development of the course curriculum, which has been published previously [15], was informed by the theoretical considerations described above. These perspectives provided a conceptual basis for addressing complementary dimensions of interprofessional learning at the macro, meso, and micro levels. In the present study, the CIHC competency framework, SIT, and TLT were used to further operationalise these dimensions in the evaluation of the course, particularly in the development of the questionnaire. 

#### Questionnaire

Within the research team, a questionnaire consisting of 26-items was developed, including:

- Likert scales (e.g. 4 levels, 1 = very low/bad to 4 = very high/good),
- Single-answer and yes/no items,
- Sociodemographic data of the participants.

The questionnaire was developed based on the learning objectives of the curriculum and the research questions in consultation with the Central Evaluation and Accreditation Agency Hannover (ZEvA) (see Appendix 1). The development of the questionnaire was informed by the competency framework, SIT and TLT. In this process, items were systematically developed to assess competencies (macro level), professional identities and social dynamics (meso level), and personal transformation and reflection (micro level) in the fields of palliative care and interprofessional collaboration. In addition, several general questions concerning the course programme and organisation, as well as students’ interaction with other students, were included. The questionnaire was piloted with medical and social work students who were not participating in the course. The pilot test resulted in only minor linguistic refinements to the questionnaire. The survey was made available on the final day of the course via QR code as an online survey using the software SurveyMonkey. To this end, a time span was allocated at the end of the course for completion of the questionnaire, to which all 25 participating course members were invited.

#### Development of the interview guide

Within the research team, a structure and guide for the focus group discussion were developed based on the survey findings. The interview guide focused on:

1. the structure and design of the course;
2. the development of students’ knowledge, skills, attitudes, and interprofessional collaboration; as well as
3. opportunities for improvement.

Each topic block was initiated with an open-ended introductory question and subsequently explored in depth through targeted follow-up questions (see Appendix 2).

#### Focus group discussions

Two focus groups were carried out in June 2025 on different days at Hochschule Hannover with students of the course, who had consented to participate and were available at the proposed dates. Sessions were moderated by FG, who is experienced with qualitative interviews and focus groups. She did not participate in the course as an instructor and was not familiar to the participating students. The participants were provided with the results of the written evaluation in advance via email. As a period of almost two months elapsed between the final course date and the commencement of the focus group, the course content was presented at the outset of the focus group. The focus groups were initiated with an introductory phase, during which the participants introduced themselves.

Written consent was taken at the start of each focus group. Discussions were audio-recorded and transcribed verbatim. Field notes were recorded as reflections by the focus group moderator. The focus groups were conducted at a time when assessment and grading, as well as notification of successful course participation, had already been completed. Since all students who had previously expressed their willingness to participate in focus groups had subsequently done so, it was deemed unnecessary to hold any additional focus groups.

### Data analysis

#### Questionnaire

The quantitative data from the questionnaire were analysed using descriptive statistics (frequencies, means, and standard deviations). Of the 26 questionnaire items, 18 that were relevant to the research question were included in the analysis. Questions regarding participants’ interaction with other students in the course were excluded from the evaluation, which was conducted using IBM SPSS Statistics 30.

#### Focus groups

Data were analysed using inductive and deductive coding according to Kuckartz and Rädiker’s qualitative content analysis, utilising MAXQDA 2024 qualitative data analysis software [22]. An initial coding system was developed deductively based on the focus group guide. The deductive codes comprised: (a) structure and design of the course, (b) development of competences, and (c) opportunities for improvement. First, the transcript of the first focus group was coded by FG, reviewed by SvS, and revised in a joint workshop of both researchers. During this workshop, inductive codes were developed and assigned thematically to the main deductive codes. Second, the transcript of the second focus group was coded using the revised coding system, which was further expanded with new inductive codes where appropriate. Finally, the coding system was reviewed by both researchers. Field notes were considered during the interpretation of the transcripts and the development of the codes. Any discrepancies were discussed until consensus was reached.

## Results

### Questionnaire

#### Participants of the survey

A total of 19 students (out of 25 enrolled in the course) completed the written evaluation. Of these, 16 identified as female (84.2%), and 9 were studying social work (47.4%). The median age of participants was 23 years (range: 20–38). Students, who did not participate or complete the survey came from medicine (*n* = 1), nursing studies (*n* = 4) and religious education (*n* = 1) (see Table 1).

**Table 1:** Demographic profile of the survey participants (*n* = 19 students)

Variable	Number of participants *n* (%)
Gender	19 (100%)
Female	16 (84.2%)
Male	3 (15.8%)
Age	18 (100%)
20–25	12 (66.7%)
26–30	4 (22.2%)
> 30	2 (11.1%)
Degree programme	19 (100%)
Medicine	3 (15.8%)
Nursing studies	3 (15.8%)
Social work	9 (47.3%)
Religious education and social work	4 (21.1%)
Term of studying	19 (100%)
1–3	3 (15.8%)
4–6	13 (68.4%)
7–10	3 (15.8%)
Finished vocational education (*n* = 19)	
Yes	8 (42.1%)
No	11 (57.9%)
Professional experience (*n* = 19)	
Yes	14 (73.7%)
No	5 (26.3%)

#### Results of the survey

The items surveyed were assessed using a 4-point Likert scale, ranging from 1 (very low/bad) to 4 (very high/good). The added value of interprofessional collaboration was clearly recognised by all participants (4.0). From the students’ perspective, the most important learning were ‘*social and communication skills*’ (3.63), ‘*knowledge from other professional groups*’ (3.42), ‘*team-oriented problem solving*’ (3.36), and ‘*knowledge of advance care planning*’ (3.31). Additionally, the practical orientation (3.52) was rated positively (see Table 2).

**Table 2:** Development of competences

Item No.	Variable	Mean (SD)
F8.1	To what extent did your overall knowledge of palliative care increase?	3.10 (0.57)
F8.2	To what extent did your knowledge of healthcare structures increase?	3.10 (0.74)
F8.3	To what extent did your knowledge of advance care planning increase?	3.31 (0.75)
F8.4	To what extent did your knowledge of dying and grieving processes increase?	2.73 (0.81)
F9.1	To what extent did your skills in professional communication improve?	2.57 (1.02)
F9.2	To what extent did your competence in ethical decision-making increase?	2.68 (0.67)
F10.1	To what extent was your self-reflection promoted?	2.73 (0.93)
F11.1	To what extent did your knowledge of other professions increase?	3.42 (0.60)
F11.2	To what extent did your knowledge of your own role in interprofessional collaboration increase?	2.78 (0.97)
F11.3	To what extent have you benefited from the knowledge of other professions?	3.31 (0.67)
F12.1	How intensively were you able to combine methodological thinking from different professions?	2.78 (0.53)
F13.4	To what extent have you and your profession been enriched by exchanges with other professions?	3.47 (0.61)
F13.6	To what extent do you see added value in collaborating with other professions?	4.00 (0.00)
F14.1	Overall, I find the structure of the newly developed interprofessional course to be …	3.42 (0.60)
F14.2	I find the fit between the course concept and the examination format to be…	3.36 (0.68)
F14.3	The practical teaching approach adopted by the lecturers was …	3.52 (0.61)
F15.1	To what extent did the storyline method promote social and communication skills?	3.63 (0.49)
F15.2	To what extent did the Storyline Method enhance your team-oriented problem-solving skills?	3.36 (0.49)

4-point Likert scale (1 = very low/bad, 2 = low/bad, 3 = high/good, 4 = very high/good); an additional “no answer” option was provided.

### Focus groups

#### Participants

A total of 10 students participated in the focus groups. The majority of participants identified as female (*n* = 8). The mean age of participants was 22 years (range: 20–38). The focus groups lasted an average of 90 minutes each. The first focus group consisted of two students studying social work and religious education. The second focus group consisted of eight students (*n* = 2 medicine, *n* = 1 nursing, *n* = 2 social work, *n* = 3 religious education) (see Table 3).

**Table 3:** Demographic profile of focus group participants (*n* = 10 students)*

Variable	Number of participants *n* (%)
Gender	
Female	8 (80%)
Male	2 (20%)
Age	
20–25	7 (70%)
26–30	2 (20%)
> 30	1 (10%)
Degree programme	
Medicine	2 (20%)
Nursing studies	1 (10%)
Social work	3 (30%)
Religious education and social work	4 (40%)
Term of studying	
1–3	1 (10%)
4–6	8 (80%)
7–10	1 (10%)
Finished vocational education	
Yes	1 (10%)
No	9 (90%)
Professional experience	
Yes	5 (50%)
No	5 (50%)

^*^All participants of the focus groups did also participate in the survey.

#### Results of the focus groups

The analysis of the focus groups resulted in eleven sub-codes, which were allocated to the existing deductive codes (see Table 4). The sub-codes emerged from open coding and were partially further differentiated at a third coding level. The main codes and sub-codes are detailed below, accompanied by selected quotes.

**Table 4:** Code tree

Main codes	Sub-codes
Structure and design of the course	Organisation
	Time planning
	Methods
Development of competences	Knowledge
	Skills
	Attitudes
	Interprofessional collaboration
*Opportunities for improvement*	Organisation
	Exchange among students
	Practical orientation
	Supplementation and deepening of content

##### Structure and design of the course

The structure and design of the course is differentiated in (a) organisation, (b) time planning, and (c) methods.

According to the participants, the course followed a common thread and was well structured. The students were divided into small interprofessional groups, which developed their own case studies during the course. However, medical students were underrepresented in some groups compared to students from other disciplines, which had an impact on interprofessional collaboration.

*I found that certain professions were underrepresented in the block course. It was very social-work-oriented*,* I would almost say. So*,* the medical and nursing aspects*,* and also pastoral care at the beginning of our group work*,* were somewhat neglected. (Stud_03)*

Conversely, it was noted that the interprofessional teaching team comprised a diverse range of professions, enabling the integration of content and attitudes from various disciplines in an authentic and competent manner.

*I especially liked the fact that we really had a team of lecturers who came from a wide variety of professions. It wasn’t just nursing and medicine saying*,* ‘Come on*,* let’s organise a seminar where we also include social workers and religious educators*,*’ but rather that the teaching team was truly interprofessional. And um*,* yes*,* you could really notice that in the implementation of the seminar. (Stud_03)*

The students held divergent opinions regarding the designated time frame for the event. The students expressed dissatisfaction with the duration of the interval between the two blocks of the course, perceiving it to be insufficient. The duration of this period was four weeks, during which the students were required to complete an assignment on ethical decision-making and to prepare a presentation.

*Those four weeks in between were also very short*,* considering that you didn’t just have to deal with the ethical dilemma in between*,* but you also had a lot more to prepare for the exam. (Stud_05)*

The time allocated for interactive group work and for exchanges with practitioners from regional hospice and palliative care was also considered insufficient.

Students expressed satisfaction with the methods used, describing them as well-aligned with the content, engaging, practical, and supportive of learning.

*The methods worked quite well for the individual elements. It did help quite a bit to introduce this specific topic*,* for example the first one*,* finding patients*,* designing it with flipcharts. Or also the emergency form*,* where you have a tactile emergency form in front of you and can see it. So*,* the methodology did help to make it a bit more realistic and practical. So that was very helpful. (Stud_05)*

In particular, the storyline method was well received, as it allows for gradual development of one’s own case.

However, the level of freedom in designing the patients and relatives was sometimes perceived as overwhelming.

*It was difficult for some professions to completely design these patients themselves*,* so to speak. It might have been easier to have a completely pre-made case and perhaps only have to think of one or two small details. (Stud_05)*

The final case presentation, which also served as the course’s examination format, received mixed evaluations from the students. Some expressed concern over the lack of clear guidelines, while others viewed the format as consistent with the seminar’s overall structure.

*I found it a bit difficult to say*,* ‘It’s your decision how you shape it [final case presentation]*,*’ but then to say in reflection*,* ‘That’s what we were missing.’ Hmm (critical). (Stud_13)*

##### Development of competences

Competency development occurred across four domains: (a) knowledge, (b) skills, (c) attitudes, and (d) interprofessional collaboration.

Participants reported an increase in profession-specific knowledge related to hospice and palliative care.

*What was new to me were the various services offered in hospice care and palliative care: inpatient hospices*,* options for care at home*,* and specialised outpatient palliative care teams – in short*,* the whole subject of palliative care. (Stud_05)*

However, the increase in profession-specific knowledge was also perceived as limited and insufficient. Some students expressed a desire for more in-depth coverage of profession-specific content and greater opportunities to acquire profession-specific skills.

*I think the problem with the knowledge was that they tried to keep it as general as possible because they didn’t want to go into too much detail for the individual professions. But for the profession itself*,* it was kind of rubbish because we already knew that stuff. And for the other professions*,* it didn’t really add much value either*,* because it was still theoretical and you couldn’t really do much with it. (Stud_08)*

Students expressed uncertainty regarding the extent to which they were able to develop practical skills. While they reported improvements in communication skills, they did not necessarily perceive further development of other specific skills.

*I would say that I haven’t directly acquired or learned any new skills. Perhaps I would say that I have refined them*,* rather. (Stud_14)*

Instead, students reported an expansion and refinement of their perspectives on the care of patients in palliative care settings, accompanied by the development of a more systemic and holistic approach to treatment. The most diverse competency developments were reported at the levels of attitudes and interprofessional collaboration.

*We worked with other professions and then reflected on how we interacted with these professions. […] I reflected on my profession in this team*,* and I reflected on my interaction with patients. I think it was a kind of reflection that we hadn’t really had before*,* because we had never worked with patients before*,* I mean*,* real patients*,* so it was something new. And even in this interprofessional team*,* you first must reflect on what my role is*,* what my task is. What are the others’ tasks? Where do I need to take a step back? Where do I need to act stronger? (Stud_05)*

Participants recognised the importance of interprofessional collaboration in palliative care and the need to engage in dialogue with other professional groups, even when perspectives and attitudes differ.

*You really have to cooperate with each other and not look at things individually. Because I can’t decide when a person will be discharged*,* but I can plan what happens after discharge. The doctor can’t do that because it’s not her job. So*,* I believe that the essential thing is to really work together*,* to go hand in hand*,* and not for everyone to run around in their own parallel worlds. (Stud_14)*

During the course, participants recognised both the added value and the limitations of their own professions in end-of-life care.

##### Opportunities for improvement

Potential for improvement was identified in the areas of (a) organisation, (b) exchange among students, (c) practical orientation, as well as (d) the supplementation and deepening of content.

Students expressed a preference for more time between the two course blocks and closer supervision.

*I think the course structure was pretty good. I liked that it was divided into two blocks with plenty of time in between. But I must also say that the four weeks in between were very intense. Considering that it was in the middle of the semester*,* (…) it might have been useful to have a meeting with the lecturers in between. (Stud_03)*

Students were given a high degree of creative freedom in designing their cases. Some expressed a desire for a stronger focus on additional topics and a more structured case design guided by the lecturers.

Regarding interprofessional exchange, students also expressed a desire for increased interaction within their own professional groups.

*I think it’s a good idea to consult within the professions. I believe that would also convey much*,* much more certainty for interprofessional cooperation for the respective profession. (Stud_08)*

The proposal involves incorporating profession-specific learning units aimed at promoting the acquisition of profession-specific skills. To foster interprofessional cooperation, the students would like to see a balanced representation from all degree programmes within the course.

In addition, students expressed a desire for a stronger practical focus, for example through visits to palliative care facilities and hospices. Furthermore, students expressed a desire for direct contact with patients during the course and the opportunity to apply and test the content they had learned with actual patients.

*I just missed more practical orientation. It was all very theory-oriented and imaginary. There were no patients*,* no real ones. I thought at the beginning of this seminar that we would be working with real patients. We didn’t. That was a bit disappointing. (Stud_07)*

According to students, the course content primarily focused on topics assumed to be related to social work, such as communication, ethical decision-making.

*Social work was very prominent in the first block. (...) They could have cut back a bit there. Even though I represent social work and am always in favour of others learning something about my profession*,* I can also understand that it simply went a bit beyond the scope (...) in relation to the other professions. (Stud_03)*

Students also expressed a desire to explore topics such as ethics, care structures, and end-of-life rituals in greater depth.

## Discussion

This study presents findings from the second implementation cycle of an innovative interprofessional curriculum in palliative care [15], designed to foster collaborative competence through narrative-based, experiential learning.

### Main findings

Our findings suggest that the course supported students’ interprofessional collaboration and understanding of holistic palliative care. While reflective practice received a comparatively lower rating in the written evaluation (mean 2.73), reflection on professional roles and interprofessional interactions emerged more prominently in the focus groups conducted two months after the course. This difference may indicate that some aspects of reflective learning become more salient over time; however, as the study was not designed to assess longitudinal outcomes, the sustainability of these learning effects cannot be established. Several challenges also emerged, particularly concerning the balance of professional representation, the depth of discipline-specific content, and the integration of real-world clinical experiences.

### Theoretical integration

The study’s alignment with the three-tiered theoretical framework – macro (CIHC), meso (SIT), and micro (TLT) – proved instrumental in interpreting the complexity of interprofessional learning.

At the macro level, participants reported significant gains in core interprofessional competencies, particularly in communication, team functioning, and patient-centred care. These findings resonate with existing literature showing that structured IPE programs improve learners’ ability to work collaboratively across professions [23]. The high ratings for ‘*social and communication skill’* and ‘*team-oriented problem solving*’ suggest that the storyline method effectively simulated real-world team dynamics, allowing students to practice role clarification and collaborative decision-making in a safe, reflective environment.

At the meso level, the application of SIT offers valuable insights into the dynamics of interprofessional group work. While the course fostered positive intergroup contact through a diverse teaching team and shared narrative tasks, the underrepresentation of medical and nursing students in some groups – despite their central role in palliative care – may have inadvertently reinforced professional hierarchies. The students’ positive perceptions of the interprofessional teaching team can also be interpreted through the lens of observational learning. Observing professionals from different disciplines working together may have provided students with opportunities to observe and reflect on collaborative behaviours that complemented the explicit curriculum [24, 25]. Such modelling may provide opportunities for reflection on professional roles and identities, as students learn not only through formal instruction but also by observing how professional roles and relationships are enacted [25, 26]. From the perspective of interprofessional socialisation, these interactions may also contribute to the development of a dual identity, in which students maintain their professional identity while developing a sense of belonging to an interprofessional team [27]. The students’ reflections on their own professional roles and on when to step back or take a more active role suggest that the course created opportunities to negotiate both professional and interprofessional identities. However, the unequal representation of professions may have limited opportunities for some students to engage in interprofessional socialisation and to develop a sense of dual identity. As one student noted, the course felt “*very social-work-oriented*”, which may have triggered in-group favouritism or marginalisation of other professional identities. This finding underscores a critical challenge in IPE: even well-intentioned curricula risk to create power imbalances if professional representation and content are not carefully balanced [28, 29]. The observed desire for more equitable participation across disciplines is consistent with previous research highlighting the value of integrating diverse disciplines into interprofessional education [30] and underscores the importance of intentional recruitment and group composition strategies [31].

At the micro level, the findings suggest that the course provided opportunities for reflective learning. Students reported reflections on their professional roles, responsibilities, and interactions with other professions, which can be interpreted through the lens of TLT. The narrative approach, by embedding learners in the perspective of a fictional patient and relatives, provided opportunities for reflection on professional roles and interactions with other professions. As one student reflected: “*I reflected on my profession in this team… where do I need to take a step back?*” This reflection on professional roles and boundaries is particularly relevant in palliative care, where professional identity is closely intertwined with values of dignity, presence, and ethical sensitivity [32]. Although ethical decision-making was addressed within the course, it received a lower rating in the written evaluation (mean 2.68) than social and communication skills (mean 3.63). This may indicate that ethical decision-making was perceived as a less prominent area of competence development, which is consistent with students’ expressed desire to explore ethical aspects in greater depth.

### Addressing gaps in interprofessional palliative care education

Our findings confirm and extend previous research on IPE in palliative care. Several IPE programs demonstrated improvements in learners’ knowledge and attitudes, although study designs and outcome measures were variable [23]. Only few studies have systematically addressed the underrepresentation of non-medical professions – particularly social work, chaplaincy, and pharmacy – or the systematic integration of spiritual care into educational curricula [4, 5]. Yet, our data show that students perceived the course as overly focused on social work and ethics. Some systematic reviews of palliative care education note that many curricula place strong emphasis on communication and psychosocial domains, with clinical content and longer-term competency outcomes less consistently addressed: a pattern that may influence how learners from medicine and nursing perceive the relevance of such programs [33].

Moreover, the students’ expressed desire for direct patient contact, visits to hospices, and practical immersion reveals a critical gap between simulation-based learning and authentic clinical experience. While the storyline method is a powerful tool for fostering empathy and narrative competence, it cannot fully replace real-world encounters with patients and families. As one student lamented: “*We didn’t have real patients… that was a bit disappointing.*” This sentiment echoes findings from other studies showing that learners value experiential learning with actual patients [34, 35]. Future iterations of the course should reflect time limitations, balance narrative engagement with discipline-specific depth and integrate structured clinical placements or simulated patient encounters to fully prepare students for the complex, collaborative realities of palliative care practice.

### Implications for curriculum design and future research

The study’s results offer concrete recommendations for improving interprofessional palliative care education:

1. Ensure balanced professional representation in both student groups and teaching teams to prevent dominance by any single profession and to foster equitable collaboration.
2. Integrate discipline-specific learning units within the overarching narrative framework to ensure that all professions develop relevant clinical and practical skills.
3. Incorporate real-world experiences, such as visits to palliative care units, hospices, or home care settings, to enhance practical orientation and emotional engagement.
4. Strengthen interprofessional supervision with regular check-ins between course blocks to support students during the reflective phase.

Finally, this study contributes to the growing body of evidence that IPE in palliative care must go beyond competence development to include personal transformation and ethical reflection. Future research should explore longitudinal outcomes, such as changes in professional identity, career choices, or clinical practice, following participation in such programs. Additionally, qualitative studies could further investigate how students from different professions experience and negotiate their identities within interprofessional teams.

### Strengths and limitations

This study benefits from a robust mixed-methods design that combines quantitative survey data with in-depth qualitative focus group analysis, enabling a comprehensive and nuanced understanding of students’ learning experiences. The use of the Consolidated Criteria for Reporting Qualitative Research (COREQ) ensures transparency and rigor in the qualitative analysis, while the integration of Kuckartz and Rädiker’s qualitative content analysis with both inductive and deductive coding enhances the credibility and depth of the findings. The study’s theoretical grounding in the CIHC framework, SIT, and TLT allows for a multi-level interpretation of interprofessional learning, moving beyond surface-level outcomes to explore professional identity formation, relational dynamics, and personal transformation; dimensions often underemphasised in IPE research.

A further strength is that this study represents the second implementation cycle of the curriculum, allowing the research team to build on experiences and insights from the first implementation cycle and to further develop and evaluate the course.

A key strength lies in the interdisciplinary composition of the research and teaching team, which included experts from medicine and medical didactics, nursing science and nursing, social work, religious education, and social sciences. This diversity not only enriched the curriculum development but also ensured that the evaluation process was informed by multiple professional perspectives, reducing potential bias and enhancing the validity of the findings.

However, the relatively small sample size and the composition of the focus groups represent limitations. The first focus group consisted of only two students from the fields of social work and religious education, with no medical or nursing students represented in this group. This uneven representation may have influenced the interprofessional dynamics and the range of perspectives represented in the focus groups. In addition, none of the focus group participants had previous professional experience. As participation in the focus groups was voluntary and reasons for participation were not systematically collected, it remains unclear why students without previous professional experience were more strongly represented.

Furthermore, the course’s use of the storyline method as a narrative, experiential, and collaborative pedagogical approach provided a unique learning environment that fostered empathy, creativity, and reflective practice, particularly in the emotionally complex domain of palliative care. The lack of direct patient contact during the course, while acknowledged as a limitation by students, also means that the study cannot assess the transfer of learning into real clinical practice. This is a limitation of the study. 

Finally, the two-month interval between course completion and focus group discussions, while intended to allow for reflection, may have led to memory decay or shifting perceptions, potentially affecting the depth of recall.

## Conclusions

In conclusion, this study demonstrates that a theoretically grounded, narrative-based interprofessional curriculum can effectively promote collaborative competence, reflective practice, and holistic understanding in palliative care education. By integrating CIHC, SIT, and TLT, the course successfully addressed multiple levels of learning. However, challenges related to professional balance, content depth, and practical immersion must be addressed to ensure inclusivity, relevance, and authenticity. As palliative care becomes increasingly team-based and patient-centred, so too must its education evolve, toward models that are not only interprofessional but also transformative, ethical, and grounded in real-world experience.

## Supplementary Information

Supplementary Material 1.

Supplementary Material 2.

Supplementary Material 3.

## Data Availability

The datasets used and analysed during the current study are available from the corresponding author on reasonable request.

## Abbreviations

CIHC: Canadian Interprofessional Health Collaborative framework
IPE: Interprofessional Education
SIT: Social Identity Theory
TLT: Transformative Learning Theory
