# Supplementary material for: Interprofessional education in palliative care: a theory-informed evaluation of a storyline-based curriculum for health and social care students
Source: BMC Med Educ. 2026 Oct 22;26:1702. doi: 10.1186/s12909-026-10663-3 (PMC13689147; doi:10.1186/s12909-026-10663-3)
Supplement: Supplementary file 2 [file 12909_2026_10663_MOESM2_ESM.docx]

**Appendix 2: Interview guide**

**Study objective:**

The aim of the study is to evaluate and ensure the quality of the course “Interprofessional Collaboration in Palliative Care” for students of nursing, medicine, social work, and religious education.

**Research questions:**

1) What is the students' perspective on the structure and design of the course?

2) How has the course affected students' development of competences in interprofessional collaboration and palliative care?

3) What opportunities for improvement do students see on the course?

**Semi-structured discussion guide:**

**Domain 1: Structure and design of the course**

**Key question:**

Over the next 40 minutes, we would like to talk about your views on the structure and design of the course "Interprofessional Collaboration in Palliative Care."

Please tell us how you experienced the **structure and design of the course**.

**Follow-up questions:**

- How did you perceive the organization of the course?
- How did you perceive the timing of the course?
- How did you perceive the method of case development (storyline) that was used?
  - How helpful were the individual methods for your learning process?
  - How did you perceive the collaboration between the different professions in the small group?
  - How did you perceive the communication within the small group?
  - What went well?
  - What challenges did you encounter?
- How did you perceive the case presentation at the end of the seminar as a form of assessment?
- Were there any aspects of the structure and design of the course that you felt were not appropriate, and how should they be changed to increase learning success and quality of the course for future participants?

**Domain 2: development of competences**

**Key question:**

In the next 40 minutes, we want to talk about your view of your competence development through the course "Interprofessional Collaboration in Palliative Care."

Please tell us how your competencies in interprofessional collaboration and palliative care have changed as a result of the course.

**Follow-up questions:**

- What were the most important insights you gained from the course?
- What specific things did you learn about palliative care that you didn't know before?
- What specifically did you learn about interprofessional collaboration that you did not know before?
- What skills and methods did you acquire through the course, and to what extent?
- To what extent have you been able to apply what you have learned?
- To what extent has your ability to self-reflection changed?
- How has the course changed your view of working with other professional groups?
- To what extent has the course helped you to better understand the perspectives of other professions?
- How have your attitudes or preconceptions toward other professional groups changed as a result of the course, and why?
- What changes to the course content do you consider useful in order to better promote development of competences for future participants?
  - Were there any aspects of the content that you think could be shortened or omitted?
  - Were there any aspects of the content that you felt were missing?

**Final questions**

- Do you have any further ideas or suggestions on how the seminar could be made even more valuable for future participants?
- What thoughts and feelings arise when you think back on the seminar?
