# Supplementary material for: Interprofessional education in palliative care: a theory-informed evaluation of a storyline-based curriculum for health and social care students
Source: BMC Med Educ. 2026 Oct 22;26:1702. doi: 10.1186/s12909-026-10663-3 (PMC13689147; doi:10.1186/s12909-026-10663-3)
Supplement: Supplementary file 3 [file 12909_2026_10663_MOESM3_ESM.docx]

**Appendix 2: Questionnaire**

F1

Please enter your current age in years

_______________

F2

Please indicate your gender

| female | ⃝ |
| --- | --- |
| male | ⃝ |
| divers/queer | ⃝ |
| No answer | ⃝ |

F3

What subject are you studying?

| Medicine | ⃝ |
| --- | --- |
| Nursing | ⃝ |
| Social Work | ⃝ |
| Religious Education | ⃝ |

F4

Which semester are you studying in?

_______________

F5

Have you already completed another degree programme?

| No | ⃝ |
| --- | --- |
| Yes | ⃝ |
| If „yes“, which? | ______________ |

F6

Have you already completed a vocational training?

| No | ⃝ |
| --- | --- |
| Yes | ⃝ |
| If „yes“, which? | ______________ |

F7

Do you have any previous work expirience?

| No | ⃝ |
| --- | --- |
| Yes | ⃝ |
| If „yes“, which? | ______________ |

F8

How much did your knowledge increase in relation to…

|  | very  low | low | high | very  high | no answer |
| --- | --- | --- | --- | --- | --- |
| … Palliative Care at all? | ⃝ | ⃝ | ⃝ | ⃝ | ⃝ |
| … service structures? | ⃝ | ⃝ | ⃝ | ⃝ | ⃝ |
| … Advance Care Planning? | ⃝ | ⃝ | ⃝ | ⃝ | ⃝ |
| … dying and bereavement  processes? | ⃝ | ⃝ | ⃝ | ⃝ | ⃝ |

F9

Increased **skills/ methods** in Palliative Care …

|  | very  low | low | high | very  high | no answer |
| --- | --- | --- | --- | --- | --- |
| How much did your skills in professional communication improve? | ⃝ | ⃝ | ⃝ | ⃝ | ⃝ |
| How much did your competence in ethical decision-making increase? | ⃝ | ⃝ | ⃝ | ⃝ | ⃝ |

F10

Development of your **attitude**:

|  | very  weak | weak | strong | very strong | no answer |
| --- | --- | --- | --- | --- | --- |
| How strongly was your self-reflection encouraged? | ⃝ | ⃝ | ⃝ | ⃝ | ⃝ |
| How strongly was your openness encouraged? | ⃝ | ⃝ | ⃝ | ⃝ | ⃝ |
| How strongly was your empathy encouraged? | ⃝ | ⃝ | ⃝ | ⃝ | ⃝ |
| How much did the course contribute to your personal development? | ⃝ | ⃝ | ⃝ | ⃝ | ⃝ |

F 11

Knowledge about **interprofessional collaboration:**

|  | very  weak | weak | strong | very strong | no answer |
| --- | --- | --- | --- | --- | --- |
| How much did your knowledge increase of other professions? | ⃝ | ⃝ | ⃝ | ⃝ | ⃝ |
| How much did your knowledge increase of your own role in interprofessional collaboration? i | ⃝ | ⃝ | ⃝ | ⃝ | ⃝ |
| How much have you benefited from the knowledge of others professions? | ⃝ | ⃝ | ⃝ | ⃝ | ⃝ |
| How much did you contribute your professional knowledge? | ⃝ | ⃝ | ⃝ | ⃝ | ⃝ |

F12

**Skills/methods** in **interprofessional collaboration:**

|  | very  weak | weak | intense | very  intense | no answer |
| --- | --- | --- | --- | --- | --- |
| How intensively have you familiarized yourself with the methodological approaches of other professions? | ⃝ | ⃝ | ⃝ | ⃝ | ⃝ |
| How intensively were you able to combine methodical thinking from different perspectives? | ⃝ | ⃝ | ⃝ | ⃝ | ⃝ |

F 13

**Attitude** in **interprofessionel collaboration**:

| **To what extent …** | very  low | low | extensive | very extensive | no answer |
| --- | --- | --- | --- | --- | --- |
| … are you able to openly express your attitudes, opinions and beliefs? | ⃝ | ⃝ | ⃝ | ⃝ | ⃝ |
| … have you learned new and different attitudes? | ⃝ | ⃝ | ⃝ | ⃝ | ⃝ |
| … did the attitudes of the other participants encourage you to reflect and explore your feelings? | ⃝ | ⃝ | ⃝ | ⃝ | ⃝ |
| … have you and your profession been enriched by exchanges with other professions? | ⃝ | ⃝ | ⃝ | ⃝ | ⃝ |
| … did you find working with other professions challenging? | ⃝ | ⃝ | ⃝ | ⃝ | ⃝ |
| … do you see added value in collaborating with other professions? | ⃝ | ⃝ | ⃝ | ⃝ | ⃝ |

F 14

General information about the course concept

|  | yery poor | poor | good | very  good | no answer |
| --- | --- | --- | --- | --- | --- |
| I think the expansion of the newly developed course is … | ⃝ | ⃝ | ⃝ | ⃝ | ⃝ |
| I think the course concept and course format are matched … | ⃝ | ⃝ | ⃝ | ⃝ | ⃝ |
| The practical teaching of learning content by the instructors was … | ⃝ | ⃝ | ⃝ | ⃝ | ⃝ |
| How well did the teachers coordinate within the team? | ⃝ | ⃝ | ⃝ | ⃝ | ⃝ |

F15

The **storyline method**

|  | very  weak | weak | strong | very  strong | no answer |
| --- | --- | --- | --- | --- | --- |
| To what extent did the storyline method promote social and communication skills? | ⃝ | ⃝ | ⃝ | ⃝ | ⃝ |
| To what extent did the method promote their team-oriented problem-solving skills? | ⃝ | ⃝ | ⃝ | ⃝ | ⃝ |

F16

**Interprofessional collaboration in the course**

|  | very  weak | weak | stong | very  strong | no answer |
| --- | --- | --- | --- | --- | --- |
| How much did you contribute to your working group? | ⃝ | ⃝ | ⃝ | ⃝ | ⃝ |
| To what extent were your contributions taken into account? | ⃝ | ⃝ | ⃝ | ⃝ | ⃝ |
| How often have you asked students from other professions about their perspectives? | ⃝ | ⃝ | ⃝ | ⃝ | ⃝ |

F17

Who did you interact with outside of the small group work?

| with students from all disciplines | ⃝ |
| --- | --- |
| mainly with students of other subjects | ⃝ |
| mainly with students from my own subject | ⃝ |
| with no other students | ⃝ |

F18

Did you have any reservations about other professions before the course?

| Yes , I did | ⃝ |
| --- | --- |
| No, I didn`t | ⃝ |

F19

If you had reservations about other professions before the course, can you give some examples?

______________________________________________________________________________

F20

Has the course changed your attitude toward other professions?

| Yes, but not in a positive way. | ⃝ |
| --- | --- |
| It depends more on individual persons | ⃝ |
| No, it hasn`t changed | ⃝ |
| Yes, I have a better understanding than other profession. | ⃝ |

F21

Level of difficulty of the course

|  | far  too low | too  low | appropriate | too  high | much too high |
| --- | --- | --- | --- | --- | --- |
| Overall, I found the level of performance requirements in the course … | ⃝ | ⃝ | ⃝ | ⃝ | ⃝ |
| I found the level of performance requirements in self-study … | ⃝ | ⃝ | ⃝ | ⃝ | ⃝ |
|  |  |  |  |  |  |
| How many hours did you spend outside of class time at the university for the course? | ___________ | | | | |

F22

What did you learn the most about?

____________________________________________________________________________

F23

What was the biggest challenge in the course?

_____________________________________________________________________________

F24

Safety in dealing with those affected after the course

|  | no safer than before | barely safer than before | slightly safer than before | much safer than before | not specified |
| --- | --- | --- | --- | --- | --- |
| How confident do you feel about accompanying seriously ill and dying patients? | ⃝ | ⃝ | ⃝ | ⃝ | ⃝ |
| How confident do you feel about accompanying relatives? | ⃝ | ⃝ | ⃝ | ⃝ | ⃝ |

F25

Interest in palliative care

|  | Very  low | low | strong | very strong | not specified |
| --- | --- | --- | --- | --- | --- |
| How interested are you in continuing to work in palliative care? | ⃝ | ⃝ | ⃝ | ⃝ | ⃝ |

F26

Recommendation to other students

|  | under no circumstances | rather not | Yes, but with restrictions | Yes, definitely. | not specified |
| --- | --- | --- | --- | --- | --- |
| Would you recommend this course to other students? | ⃝ | ⃝ | ⃝ | ⃝ | ⃝ |

F27

What grade would you give the course overall? (about 0 points = 6 … to 15 points =1+)

_______________________

F28

What else I would like to say:

________________________________________________________________________________
